# Cyclin-Dependent Kinase-Like 5 (CDKL5): Possible Cellular Signalling Targets and Involvement in CDKL5 Deficiency Disorder

**DOI:** 10.1155/2020/6970190

**Published:** 2020-06-05

**Authors:** Syouichi Katayama, Noriyuki Sueyoshi, Tetsuya Inazu, Isamu Kameshita

**Affiliations:** ^1^Department of Pharmacy, College of Pharmaceutical Sciences, Ritsumeikan University, Shiga 525-8577, Japan; ^2^Department of Life Sciences, Faculty of Agriculture, Kagawa University, Kagawa 761-0795, Japan

## Abstract

Cyclin-dependent kinase-like 5 (CDKL5, also known as STK9) is a serine/threonine protein kinase originally identified in 1998 during a transcriptional mapping project of the human X chromosome. Thereafter, a mutation in *CDKL5* was reported in individuals with the atypical Rett syndrome, a neurodevelopmental disorder, suggesting that CDKL5 plays an important regulatory role in neuronal function. The disease associated with *CDKL5* mutation has recently been recognised as CDKL5 deficiency disorder (CDD) and has been distinguished from the Rett syndrome owing to its symptomatic manifestation. Because *CDKL5* mutations identified in patients with CDD cause enzymatic loss of function, CDKL5 catalytic activity is likely strongly associated with the disease. Consequently, the exploration of CDKL5 substrate characteristics and regulatory mechanisms of its catalytic activity are important for identifying therapeutic target molecules and developing new treatment. In this review, we summarise recent findings on the phosphorylation of CDKL5 substrates and the mechanisms of CDKL5 phosphorylation and dephosphorylation. We also discuss the relationship between changes in the phosphorylation signalling pathways and the *Cdkl5* knockout mouse phenotype and consider future prospects for the treatment of mental and neurological disease associated with *CDKL5* mutations.

## 1. Introduction

Protein phosphorylation is a chemical modification that plays a crucial role in many pivotal biological processes, including cell division, differentiation, and higher-order neural function. This reaction is catalysed by protein kinases. Defects in genes encoding protein kinases cause a myriad of different diseases [1–4]. Cyclin-dependent kinase-like 5 (CDKL5), a protein kinase, is the focus of this study, and its gene, *CDKL5*, has historically been linked to the atypical Rett syndrome. However, recently the disease associated with *CDKL5* deficiency was distinguished from the Rett syndrome and identified as a unique disorder called “CDKL5 deficiency disorder (CDD).” In the current review, we discuss molecular- to individual-level analyses of *CDKL5* dating back to its discovery.

The Rett syndrome is an X-linked neurodevelopmental disorder first reported in the 1960s [5] and is estimated to affect 1 in every 10,000 to 15,000 live female births. Mutations in genes encoding methyl-CpG-binding protein 2 (*MECP2*), cyclin-dependent kinase-like 5 (*CDKL5*, also known as *STK9*), and forkhead box protein G1 (*FOXG1*) were thought to be the primary genetic drivers of the condition [6–8]. Because the *MECP2* mutation is the most commonly observed cause of the Rett syndrome, the disease caused by the *MECP2* mutation is called the typical Rett syndrome, whereas that involving the other genes is historically called the atypical Rett syndrome (*CDKL5* mutation, Hanefeld variant; *FOXG1* mutation, congenital variant) [9–11]. However, mutations in *CDKL5* and *FOXG1* have resulted in unique diseases that are distinguishable from the Rett syndrome, since the specific symptoms of the disease vary depending on the causative gene involved [12–14]. For example, mutations in *CDKL5* cause early life epilepsy [15, 16], while those in *FOXG1* are known to cause characteristic stereotypic movements and severe microcephaly [17, 18]. The diseases caused by *CDKL5* and *FOXG1* mutations are referred to as CDD (ICD-10-CM code; G40.42) and FOXG1 syndrome, respectively. Because an effective treatment for these diseases is yet to be found, elucidation of the molecular signalling pathways controlled by the driver genes is an important prerequisite for the development of viable therapies.

*CDKL5* (*STK9*) was first reported in 1998 as a gene encoding a novel protein kinase situated on the X chromosome. It was discovered during a transcriptional mapping project [19]. In 2003, the relationship between *CDKL5* mutations and X-linked neurodevelopmental disorders was reported [20]. From 2004 onwards, reports of *CDKL5* as the causative gene of atypical Rett syndrome began to emerge [7]. Many new mutations have been discovered in patients with CDD so far; both missense and nonsense mutations in the *CDKL5* gene have been reported [21–25]. As most of the missense mutations occur in the region encoding the catalytic domain of CDKL5, it is believed that reduced activity of the enzyme—specifically, its ability to modify amino acid residues—is the direct cause of the onset of disease. Conversely, nonsense mutations in *CDKL5* result in the formation of proteins lacking the C-terminus. The C-terminal region contains a sequence vital to the regulation of the intracellular localisation of CDKL5, and the mutations result in abnormal CDKL5 localisation patterns, as observed in an *in vitro* transient expression study [26]. Furthermore, nonsense mutations completely inhibit the ability of CDKL5 to interact with proteins that typically interact with its C-terminus. These observations illustrate the possible mechanisms of the involvement of nonsense mutations in disease onset. However, it is also possible that CDD is caused by the loss of *CDKL5* expression if a premature stop codon triggers nonsense-mediated decay (NMD) of *CDKL5* mRNA. CDKL5 fragment was not detected in the R59X nonsense mutation knockin mice [27], indicating the occurrence of NMD.

Based on the above, CDKL5 is an important protein kinase strongly associated with CDD. Recently, much research has been dedicated to this protein, including target exploration and *in vivo* knockout analysis in mouse models. In the current review, we will outline the major findings of CDKL5 research and discuss possible future developments.

## 2. *CDKL5* and Its Transcripts

In 1998, *STK9*, which codes for a serine/threonine (Ser/Thr) protein kinase, was cloned during a transcriptional mapping project of the Xp22 chromosomal region [19]. Because the kinase domain of *STK9* shares high similarity with that of enzymes encoded by the *CDK* gene family [28], the gene was later renamed *CDKL5*. The length of the human *CDKL5* is 228 kb, and the gene contains 27 exons. Reports of differential splicing indicate that various isoforms of the protein exist, including one that is strongly expressed in the brain and another expressed abundantly in the testes [29–31].

**Figure 1:**
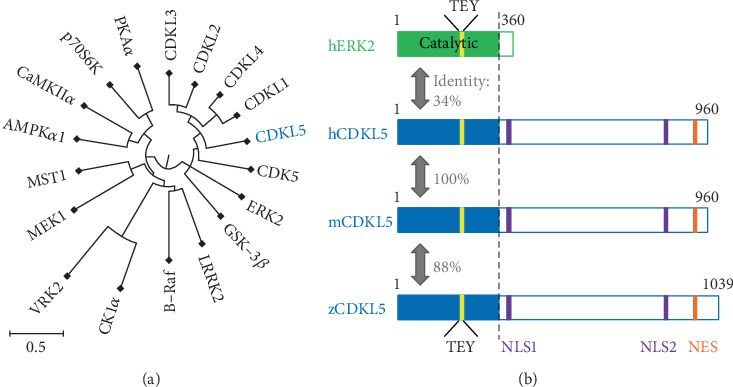
Primary structure and molecular phylogenetic tree of CDKL5. (a) Circular phylogenetic trees for the catalytic domains of CDKL5 and various kinases were generated using the CLC sequence viewer version 8.0. Scale bars indicate substitution rates of amino acids (50% amino acid substitution rate per bar length). (b) Schematic illustration of the primary structure of CDKL5 from human (accession no. NP_001310218.1), mouse (mCDKL5_1 in Hector et al. [29]), and zebrafish (accession no. NP_001124243.1) and of ERK2 from human (accession no. NP_620407.1). The catalytic domains of enzymes have been coloured blue and green, respectively. The TEY sequence corresponding to the activation loop is shown in yellow, the nuclear translocation sequence in purple, and the nuclear export sequence in orange. Amino acid identity between each catalytic region is shown.

The *CDKL5* gene product is a Ser/Thr kinase. Its placement in the molecular phylogenetic tree of protein kinases indicates identity with the CDK and mitogen-activated protein kinase (MAPK) families (Figure 1(a)) [28]. The catalytic domain of the CDKL5 enzyme has a characteristic 12-subdomain structure of Ser/Thr kinases, the sequence of which is highly conserved across many species, from mammals to fish (Figure 1(b)) [32–34]. An activation loop, which includes the TEY sequence observed in MAPK family kinases, is present between subdomains VII and VIII of the catalytic domain. Autophosphorylation of this activation loop is well known to activate CDKL5 catalytic functions, as described in detail below. The regulatory domain at the C-terminus of CDKL5 contains two nuclear localisation signal (NLS) sequences and a nuclear export signal (NES); these signal sequences were demonstrated to regulate intracellular localisation of CDKL5 *in vitro* [35, 36].

Since CDKL5 is particularly robustly expressed in the brain and is localised in the cytoplasm and the nucleus, it is speculated to be involved in several neural functions. CDKL5 is thought to be involved in dendritic architecture, dendric spine morphology, and neuronal cell death [37–39].

## 3. Possible Targets of CDKL5

The relationship between *CDKL5* and neurodevelopmental disorders has been the subject of many reports. In particular, missense mutations in the sequence encoding the catalytic domain of CDKL5 are thought to induce CDD by causing a decline or complete loss of the enzyme function [25, 40, 41]. Consequently, exploration of the CDKL5 substrates and analysis of the biological significance of their phosphorylation will deepen our understanding of the pathology of CDD, potentially yielding significant clues for future treatment possibilities. We have collated a list of the identified CDKL5 substrates in Table 1. While the biological significance of the phosphorylation of only a small subset of these substrates has been clarified, future analyses may provide further insight, revealing previously unknown functions of CDKL5.

In 2005, Mari et al. [40, 42] reported that CDKL5 phosphorylated MeCP2 by *in vitro* kinase assay. MeCP2 regulates transcription by binding to methylated CpG sites on genomic DNA [43]. Since *MECP2* is the primary causative gene of the Rett syndrome, evaluating the biological significance of CDKL5-mediated phosphorylation of MeCP2 is important to identify the common pathology between the Rett syndrome and CDD at the molecular level. However, CDKL5 only weakly phosphorylates MeCP2 [44]. Additionally, Lin et al. asserted that MeCP2 was not directly phosphorylated by CDKL5 [41]. Therefore, the significance of phosphorylation of MeCP2 by CDKL5 remains to be clarified. In 2010, Carouge et al. [45] reported that MeCP2 suppresses CDKL5 expression. Livide et al. [46] reported that in induced pluripotent stem cells expressing either mutated MeCP2 or CDKL5, as observed in patients, the expression of glutamate D1 receptor (GluD1) protein is elevated. Based on these findings, further analysis of the association between these two proteins is warranted.

In 2008, by utilising a unique approach using Multi-PK antibodies (which can recognise multiple protein kinases) [47, 48], we discovered that the catalytic fragment of CDKL5 phosphorylates DNA methyltransferase 1 (Dnmt1) *in vitro* [49]. Since Dnmt1 preferentially methylates hemimethylated DNA, it might act to maintain genome methylation patterns across the replication cycle [50]. CDKL5 phosphorylation sites are located at the N-terminus of Dnmt1 (residues 1–290), but the specific location of these sites remains unknown. Therefore, the significance of phosphorylation of Dnmt1 by CDKL5 remains to be identified. According to previous reports, casein kinase 1 (CK1) and protein kinase C also phosphorylate the N-terminus of Dnmt1, altering its DNA-binding and methylating activity [51, 52]. Therefore, it is possible that phosphorylation of Dnmt1 by CDKL5 may also regulate its DNA-methylation ability, thereby epigenetically controlling the expression of various genes.

Further research has also shown that Ser-631 of the netrin-G1 ligand protein (NGL-1, also known as LRRC4C) is phosphorylated by CDKL5 *in vitro* [38]. NGL-1 is a membrane protein localised at the postsynaptic membrane; there, its ability to bind the presynaptic protein netrin-G1 (NTNG1) plays an important role in the formation of neural circuits [53]. Reports indicate that *NTNG1* is a causative gene of the atypical Rett syndrome [54–56], since CDKL5 has historically been considered causative gene of the atypical Rett syndrome, suggesting that NGL-1 is one of the physiological substrates of CDKL5. A neuron in which Ser-631 of NGL-1 is substituted with an Ala exhibits an abnormally elongated dendritic spine—a phenotype similar to that observed in a neuron after shRNA-mediated *CDKL5*-knockdown [38]. Based on these observations, CDKL5 phosphorylation of NGL-1 is believed to play a crucial role in the neuronal function.

**Figure 2:**
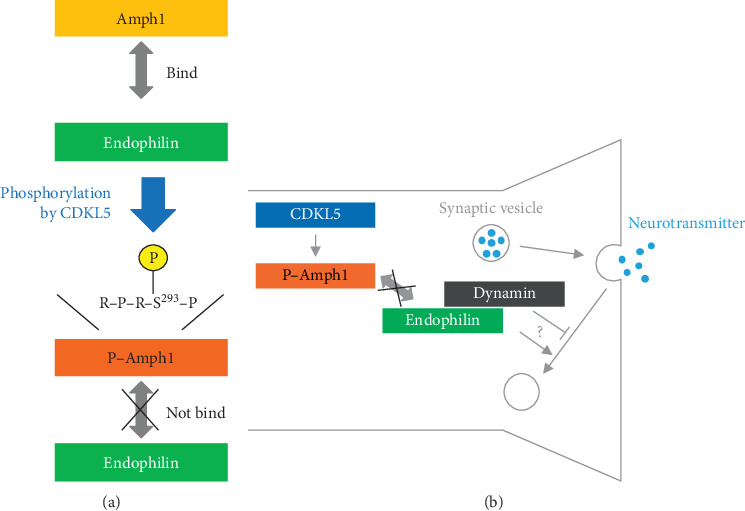
Phosphorylation of Amph1 by CDKL5 and its biological importance at the synapse. (a) Amph1 binds to endophilin, an endocytosis-related protein. Phosphorylation of a Ser-293 residue in the RPRSP sequence of Amph1 results in the loss of this binding ability *in vitro*. (b) The predicted biological importance of Amph1 phosphorylation at the synapse. At the presynaptic membrane, synaptic vesicles release neurotransmitters into the synaptic cleft and are recycled by endocytosis. A complex centred around dynamin is believed to facilitate invagination and separation of the membrane during vesicle recycling. Because Amph1 and endophilin are included in this complex, CDKL5-mediated phosphorylation of Amph1 is predicted to either positively or negatively affect membrane recycling.

In 2013, by using a unique substrate search method involving isoelectric focusing electrophoresis [57], Sekiguchi et al. discovered that Ser-293 of amphiphysin 1 (Amph1), a protein involved in clathrin-mediated endocytosis, is phosphorylated by CDKL5 *in vitro* [44]. The phosphorylation site is evolutionarily conserved; zebrafish CDKL5 phosphorylates Amph1 at this same location [32]. Because phosphorylation of Ser-293 of Amph1 inhibits its ability to bind the endocytosis-related factor endophilin, CDKL5 phosphorylation of Amph1 is thought to suppress endocytosis (Figure 2). Endocytosis plays an important role in various neuronal functions, as well as in neurodevelopment (e.g., synaptic vesicle recycling, axonal growth, and spine formation) [58–60], and loss of CDKL5 induces abnormal neuronal spine development [61]. Finally, CDKL5 binds to RAS-related C3 botulinum toxin substrate 1 (Rac1), a G-protein involved in endocytosis, and to IQGAP1, a protein that forms a complex with Rac1 [62, 63]. Therefore, it is likely that CDKL5-mediated phosphorylation of Amph1 plays an important role in spine formation.

In 2016, histone deacetylase 4 (HDAC4) was identified as an *in vitro* CDKL5 substrate [39]. By catalysing histone deacetylation, HDAC4 regulates the expression of many different genes. In addition, by responding to stimulation via the *N*-methyl-D-aspartate (NMDA) receptor, a typical glutamate receptor, HDAC4 ensures the survival of nerve cells and is involved in various aspects of their functions [64]. Typically, HDAC4 is localised in both the nucleus and the cytoplasm, and its localisation is controlled by phosphorylation [65]. Furthermore, abnormal accumulation of HDAC4 in the nucleus induces apoptosis [66]. Loss of CDKL5 also causes apoptosis [37] and enhances nuclear localisation of HDAC4 [39]. Consequently, HDAC4 might be a physiological substrate of CDKL5. The HDAC family has more than 15 members, of which proteins from classes I (HDAC1, 2, 3, and 8) and IIa (HDAC4, 5, 6, and 9) play important roles in neuronal function and survival [67]. However, only HDAC4 has been shown to be a substrate of CDKL5. Recently, however, it was reported that *HDAC8*, which plays an important role in neural differentiation, is a candidate causative gene of the atypical Rett syndrome [68, 69]. Further analysis of the relationship between HDAC family proteins and CDKL5 is needed to evaluate its importance in neuronal function and survival.

Very recently, two different research groups independently performed comprehensive screens for CDKL5 substrates and identified several candidate protein substrates [70, 71]. Among these, both groups identified microtubule-associated protein 1S (MAP1S) as a possible substrate. MAP1S binds to microtubules, regulating their stability during the cell cycle; it is also involved in autophagy [72–74]. CDKL5 phosphorylates Ser residues (Ser-786 and Ser-812 in mouse and Ser-900 in human) in the microtubule-binding domain at the C-terminus of MAP1S; phosphorylation at these sites dramatically disrupts the ability of MAP1S to bind to microtubules [71]. The life span of microtubule-associated protein RP/EB family member 3 (EB3) comets was elongated, and their distance was increased in the *Cdkl5*-knockout neuron [71]. Moreover, MAP1S knockdown reduced the comet life span increase observed in *Cdkl5*-knockout neurons. Therefore, MAP1S phosphorylation promotes microtubule dynamic instability and inhibits plus-ends growth. A study has demonstrated that CDKL5 exhibited compromised ciliogenesis [75], which may be due to microtubule instability associated with increased MAP1S phosphorylation. In addition to MAP1S, both groups also identified centrosomal protein of 131 kDa (CEP131) [70], disc large membrane-associated guanylate kinases scaffold protein 5 (DLG5) [70], and Rho/Rac guanine nucleotide exchange factor 2 (ARHGEF) [71] as an *in vitro* CDKL5 substrate. Moreover, a microtubule-associated protein RP/EB family member 2 (EB2) [71] was identified as *in vivo* CDKL5 substrates. EB2 is one of the microtubules plus-end tracking proteins that may be involved in microtubule reorganisation [76]. Therefore, it is thought that CDKL5 phosphorylates EB2 and MAP1S, respectively, and thereby contributes to microtubule dynamic instability, but the role of EB2 phosphorylation has yet to be determined.

In 2019, Fuchs et al. [77] found that CDKL5 phosphorylates SMAD3 *in vitro*. SMAD3 is a transcription factor that plays a central role in the neuronal function mediated by transforming growth factor-*β* signalling. The authors showed that protein levels of SMAD3 are reduced in the cortex and hippocampus of *Cdkl5*-knockout mouse [77]. It is therefore presumed that phosphorylation of SMAD3 regulates its protein levels. Further, CDKL5-dependent SMAD3 regulatory mechanisms control neuronal apoptotic resistance. As described above, since loss of CDKL5 accelerates apoptotic cell death [37], SMAD3 may be one of the physiological targets of CDKL5.

## 4. Mechanism of Substrate Recognition by CDKL5

One of the reasons for the scarcity of information on the protein substrates of CDKL5 is the lack of knowledge of the substrate recognition mechanism by CDKL5. The structural similarities between CDKL5 and MAPK and CDK initially prompted the researchers to consider it a proline-directed protein kinase [21]. However, using Amph1 mutants and catalytic fragment of CDKL5, we were able to determine that CDKL5 recognises the RPXS (A/P) sequence [78]. Furthermore, Muñoz et al. have confirmed that full-length CDKL5 also recognises this motif using peptide substrates [70]. Indeed, this motif is found in many CDKL5 substrates [70, 71]. Other kinases that function similarly to CDKL5 include Ca^2+^/calmodulin-dependent protein kinase II (CaMKII), which preferentially recognises an Arg residue at the P–3 position (consensus sequence: RXXS/T) [79, 80]; MAPK, which preferentially recognises a Pro residue at the P–2 position (consensus sequence: PXS/TP) [80, 81]; and dual-specificity tyrosine phosphorylation-regulated kinase 1a (Dyrk1a), which possesses both of the above specificities (consensus sequence: RPXS/TP) [82]. However, whereas these protein kinases efficiently phosphorylate substrates widely used to assess kinase activity, such as myelin basic protein and histones, CDKL5 does not appreciably phosphorylate such substrates. Furthermore, a catalytic fragment of CDKL5 does not phosphorylate metabotropic glutamate receptor 5 or septin 4, both of which contain an RPXSP sequence [78]. These observations suggest that CDKL5 recognises not only the amino acid sequence in the vicinity of the phosphorylation site but also some other external structural elements of the proteins to which it binds. By conducting a detailed analysis of the recognition mechanism using Amph1 as a model substrate, we found that the catalytic fragment of CDKL5 recognises a characteristic structural element of Amph1 known as CLAP [78]. Ser/Thr kinases are known to phosphorylate both Ser and Thr, but we have shown that CDKL5 preferentially phosphorylates the Ser residue of the substrate [78]. In addition to the RPXS (A/P) sequence at the phosphorylation site, the structural factors of the substrate are important. The details need further evaluations.

## 5. Phosphorylation of CDKL5 and Its Significance

The activity of many protein kinases is regulated through autophosphorylation and phosphorylation by upstream kinases. For example, CaMKI and CaMKIV and MAPK are activated by phosphorylation catalysed by Ca^2+^/calmodulin-dependent protein kinase kinase (CaMKK) and mitogen-activated protein kinase kinase (MAPKK), respectively [83–85]. Similarly, CaMKII is activated by autophosphorylation [86], whereas CK1 is inactivated by autophosphorylation [87]. Finally, phosphorylation of protein kinases affects their localisation, binding affinity, and substrate preference [88–90].

CDKL5 contains an activation loop characteristic for MAPK and CDK, which contains the sequence ANYTEYVAT (phosphorylated amino acids are underlined). While it is believed that these residues are autophosphorylated, whether this process is truly important for kinase activation has long remained an open question [41]. Recently, Muñoz et al. [70] have analysed the TEY motif of CDKL5 and found that substitution of the Tyr-171 residue with Ala substantially lowers CDKL5 activity *in vitro*. Nevertheless, the importance of the Thr residue phosphorylation of the TEY motif remains unknown. In 2010, Chen et al. [62] reported that CDKL5 autophosphorylates a Thr residues in response to *in vitro* stimulation by brain-derived neurotrophic factor. However, it is unclear whether this phosphorylation occurs at the Thr residue of the TEY motif or elsewhere; further analysis is needed to clarify this issue.

**Figure 3:**
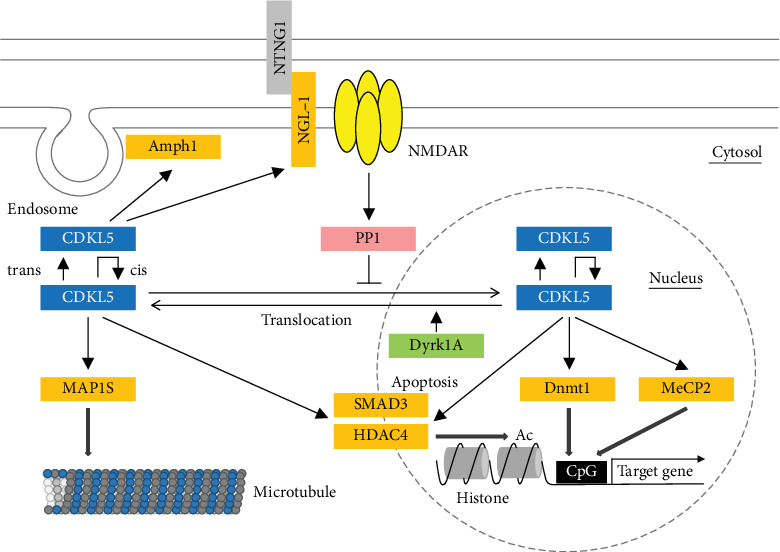
CDKL5 and phosphorylative signalling. Although CDKL5 is localised in both, the nucleus and the cytoplasm, its localisation is partly controlled by phosphorylation. Phosphorylation of CDKL5 by Dyrk1a results in its translocation to the cytoplasm. Dephosphorylation by PP1 stimulated by NMDA receptor-dependent signalling also induces strong accumulation of CDKL5 in the cytoplasm. Further, CDKL5 probably regulates its own activity via inter- and intramolecular autophosphorylation. MeCP2, Dnmt1, and HDAC4 are substrates of CDKL5, and their phosphorylation is predicted to transcriptionally regulate their control of gene expression. Finally, phosphorylation of Amph1 and NGL-1 may be involved in synapse formation and neurotransmission. Recently, phosphorylation of MAP1S has been shown to play an important role in its ability to bind to microtubules. cis: intramolecular autophosphorylation; trans: intermolecular autophosphorylation.

Since the discovery of CDKL5 in 1998, no reports detailing the phosphorylation of CDKL5 by another protein kinase have been published. However, recently, Oi et al. [36] found that Dyrk1a phosphorylates CDKL5 *in vitro*. Because *DYRK1a* is located in a region of chromosome 21 that is critical for the Down syndrome, it is believed to be closely involved in the abnormal neurodevelopment observed in that condition [3, 4]. Dyrk1a phosphorylates the Ser-308 residue of CDKL5, located near the NLS1 sequence, inhibiting CDKL5 nuclear localisation (Figure 3). CDKL5 accumulates in the nucleus (by translocating from the cytoplasm) as part of the developmental process [35], and it has been revealed that *in vitro* NMDA stimulation causes CDKL5 translocation from the nucleus to the cytoplasm [91]. The relationship between these mechanisms and Dyrk1a phosphorylation is certainly interesting and requires further study.

Little information is available regarding kinases that phosphorylate CDKL5, and phosphatases that dephosphorylate it. However, in 2015, La Montanara et al. [92] showed that CDKL5 is dephosphorylated upon *in vitro* NMDA stimulation and that this process is catalysed by protein phosphatase 1 (PP1). As mentioned above, since NMDA receptor-mediated signalling increases cytoplasmic localisation of CDKL5 in the hippocampal neuron [91], it is believed that PP1-mediated dephosphorylation confines CDKL5 to the cytoplasm (Figure 3). It is also apparent that long-term neural activity induces CDKL5 expression and degradation, and that the responsiveness of KCl stimulation is dependent on neuronal maturity *in vitro* [92], suggesting a relationship between these mechanisms of CDKL5 regulation.

To conclude, many of the mysteries surrounding the regulatory mechanisms of CDKL5 remain unsolved. In addition to the protein kinases that phosphorylate CDKL5, it is possible that the activity of CDKL5 is also regulated by binding proteins, second messengers, and even modulator proteins. The coming years should bring further insight into the mechanisms that regulate CDKL5 activity.

## 6. Pathogenic Substitutions in the Kinase Domain of CDKL5

To date, many *CDKL5* mutations have been identified in CDD patients. In particular, recent advancements in the next-generation sequencing technology have enabled highly efficient identification of mutations, and mutation databases such as the Genome Aggregation Database (gnomAD) (http://gnomad.broadinstitute.org/) and the ClinVar (https://www.ncbi.nlm.nih.gov/clinvar/) have been populated with many examples of such mutations [93, 94]. We have identified a novel *CDKL5* mutation, resulting in Y177C substitution, in a Japanese patient, and showed that it results in a complete loss of the CDKL5 enzymatic activity [25]. Nevertheless, little is known regarding the manner in which the CDKL5 modification affects the activity. Recently, we developed a method for directly measuring the activity of CDKL5 and used it to demonstrate that the activity of nearly all pathogenic CDKL5 variants is markedly reduced [95]. It is therefore believed that mutation-induced loss of CDKL5 activity plays a key role in the aetiology of CDD.

**Figure 4:**
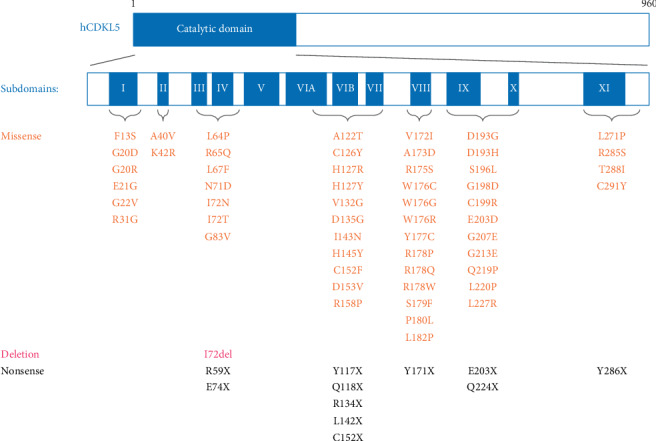
Substitutions in the catalytic domain of CDKL5. CDKL5 has an N-terminal catalytic domain, with a conserved 12-subdomain structure of Ser/Thr kinases. Sites of missense and single amino acid deletion variants extracted from RettBASE (last accessed on September 9^th^, 2019), as well as Y177C, a variant discovered by the authors of the current review, are shown.

Here, we present mutations located in the catalytic domain of CDKL5 (including the Y177C substitution), extracted from RettBASE (http://mecp2.chw.edu.au/), a database that profiles mutations in *MECP2*, *CDKL5*, and *FOXG1* [96]. After excluding all benign variants, over 50 pathogenic or likely pathogenic variants of unclear significance remained (Figure 4). Of these, a total of 11 variants are associated with nonsense mutations, and a total of 55 variants are single amino acid substitutions or single amino acid deletions. Considering the 12 subdomains of CDKL5, most of these mutations are in subdomains I–IV, or between C-terminal portion of subdomain VIA and subdomain X. Nearly no mutations have been detected between subdomain V and the first half of subdomain VIA. This suggests that subdomains I to IV and the C-terminal portion of subdomains VIA and X are particularly important for the activity of CDKL5. Because the 3D structure of the catalytic domain of CDKL5 has recently been elucidated (PDB ID: 4BGQ) [75], the molecular mechanisms through which these mutations affect CDKL5 activity might soon be revealed.

## 7. Neuronal Function of CDKL5 as Revealed by Studies Involving *Cdkl5*-Knockout Mouse

As described above, *CDKL5* is a causative gene of CDD, and many different *CDKL5* mutations have been identified. Since CDD is a neurological and mental disorder, individual-level research into the relationship between CDKL5 and the nervous system has been the primary avenue of investigation to date. Accordingly, a Cdkl5-knockout mouse model has been developed in an effort to link the CDKL5 function with the potential therapeutic targets for the disease.

Many groups have reported the analyses of *Cdkl5*-knockout mouse. In 2012, Wang et al. [97] were the first group to report on the *Cdkl5*-knockout mice, showing that loss of CDKL5 in mouse causes a characteristic clinical phenotype of CDD, including decreased fear sensitivity, impaired social behaviour, and decreased learning ability. This phenotype has subsequently been reported by many groups analysing both, *Cdkl5*-knockout and conditional *Cdkl5*-knockout mice. Motor impairment and loss of learning and/or memory are most often reported, but behaviours similar to that seen in the attention deficit hyperactivity disorder and sleep apnoea syndrome have also been observed [37, 98–105]. Thus, many analyses of *Cdkl5*-knockout mice have been reported. As conventional knockout mice, mouse models lacking exon 2, 4, or 6 have been established [97, 98, 104, 106]. Recently, R59X mutation knockin mice have also been developed [27]. Hindlimb clasping, an indicator of cerebellar ataxia, was commonly increased in exon 4-deficient and R59X knockin mice compared with wild-type [27, 98]. In addition, impairment of social behaviour by the three-chamber test is commonly observed in exon 6-deletion and R59X knockin mice [27, 97]. Interestingly, motor coordination deficiency by the rotarod test is common in exon 6-deficient mice and R59X mutant mice, but not in exon 2-deficient mice [27, 97, 104]. In addition, mice lacking exon 4 have altered kainic acid sensitivity, whereas mice lacking exon 2 show no difference in kainic acid-induced epilepsy scores [98, 106]. Such genotype-dependent phenotypic differences in mouse models are important issues for analysing the pathology of CDD.

Although many mouse models have been analysed, the mechanisms that underpin these phenotypes are still unknown. In the aforementioned paper, Wang et al. [97] found that AKT and adenosine monophosphate-activated protein kinase (AMPK) signalling is impaired by the *Cdkl5* knockout. Of these two pathways, the AKT pathway is of particular interest as a drug discovery target. Loss of CDKL5 induces abnormal activity of glycogen synthase kinase 3*β* (GSK3*β*), a molecule acting downstream of AKT, leading to insufficient neuronal maturation and an increase in the number of apoptotic cells [37]. Furthermore, treatment with GSK3*β* inhibitors SB216763 and tideglusib reportedly corrects the maturation deficiencies caused by the loss of *Cdkl5* [107, 108]. Furthermore, recently, activation of GSK3*β* has also been reported in the *Mecp2*-knockout mouse; the phenotype is partially rescued by the inhibition of GSK3*β* [109]. In a previous study, insulin-like growth factor-1 (IGF-1) was shown to alleviate some symptoms of MECP2 deficiency in mice [110]. Therefore, IGF-1-derived drugs such as trofinetide and mecasermin are highly expected as the Rett syndrome treatment drugs. IGF-1 stimulation leads to inactivation of GSK3*β* through activation of AKT [111, 112]. This also confirms that the AKT-GSK3*β* pathway is important as a therapeutic target for the Rett syndrome. CDD and the Rett syndrome have several common symptoms, and a novel common candidate drug for CDD and the Rett syndrome might be related to the AKT-GSK3*β* pathway.

One of the major symptoms of CDD is epilepsy. Recently, Okuda et al. [106] reported that *Cdkl5*-knockout mouse experiences severe NMDA-dependent epileptic seizures. As mentioned above, upon NMDA stimulation, CDKL5 is translocated from the nucleus to the cytoplasm, where this system is believed to phosphorylate proteins involved in synapse formation and neurotransmission, such as NGL-1 and Amph1. Further, CDKL5 binds to PSD-95 at the synapse and contributes to synaptic stability [113, 114]. Therefore, CDKL5 responds to NMDA and translocates to the cytoplasm, thereby possibly controlling nerve activity and network hyperexcitability. Consequently, it is believed that the function of CDKL5 in the cytoplasm is closely associated with its role in the epilepsy observed in CDD. The spontaneous epilepsy seen in CDD patients has not been observed in mouse models. In addition, as mentioned above, the epileptic response to kainic acid were dependent on the mouse model itself. Therefore, the epilepsy analysis of *Cdkl5*-knockout mice is slightly challenging and needs to be considered. However, knockout and *R59X* knockin mice showed that CDKL5 deficiency reduced 5-HT2A receptor expression in the cortex [115] and reduced 5-HT2A and GluA2, a subunit of 5-methylisoxazole-4-propionic acid (AMPA) receptor, and increased 5-HT1D expression in the hippocampus [27, 115]. Furthermore, knockout of CDKL5 has been found to enhance postsynaptic localisation of GluN2B-containing NMDA receptors in the hippocampus [106]. Therefore, knockout mice are an excellent model for analysing the association between CDKL5 and neurotransmitter receptors. If clarified, the molecular basis of the network hyperexcitability can provide important information to elucidate the mechanism of epilepsy.

## 8. Conclusions

In this review, we have described the phosphorylation signalling pathways in which CDKL5 participates and outlined the relationship between the CDKL5 signalling pathways and neurodevelopmental diseases. As CDKL5 plays many important roles in the nervous system, gene mutations leading to the loss of its activity are also believed to lead to the onset of severe neurological diseases. In practice, several pathogenic CDKL5 variants exhibit reduced or absence of phosphorylation activity [25, 41, 44]. Consequently, the identification of CDKL5 substrates is an important prerequisite for the discovery of drug targets. We have listed all heretofore-described CDKL5 substrates in Table 1, but unfortunately, the list is short. One reason for that is a lack of knowledge on the molecular mechanism of CDKL5 substrate recognition. We have demonstrated that CDKL5 binds to a characteristic domain, the CLAP domain, of Amph1 and phosphorylates the RPXS (A/P) sequence [78]. As this sequence was found in multiple substrates in recent studies [70, 71], it has been confirmed that CDKL5 prefers to phosphorylate this sequence. Nevertheless, it is still unclear whether these novel substrates contain structures corresponding to the CLAP domain of Amph1. Hence, further research into the substrate recognition mechanism of CDKL5 is necessary.

As shown in Figure 3, upstream signalling causes CDKL5 to shuttle between the nucleus and cytoplasm. Furthermore, according to recent studies, CDKL5 localisation is cell cycle-dependent [116]. Therefore, CDKL5 localisation is believed to be constantly changing. These changes allow CDKL5 to phosphorylate a variety of proteins localised in the various compartments of the cell (Figure 3). Of the CDKL5 targets, *MECP2* is particularly well known as the primary causative gene of the Rett syndrome, and its relationship with CDKL5 has been the subject of much attention. As mentioned before, *CDKL5* has historically been regarded as the causative gene of the atypical Rett syndrome. In CDD patients, seizures and sleep disturbances are more common than in the Rett patients, whereas features of regression and spinal curvature are less common [12, 15, 16]. Conversely, mental retardation and restricted or lack of speech are commonly observed in both the disorders [12, 13, 117, 118]. Therefore, an in-depth analysis of the molecular relationship between CDKL5 and MeCP2 is crucial to clarify the similarities and differences between these two disorders.

As described above, since MAP1S was identified as an *in vivo* substrate, the association between CDKL5 and microtubules has attracted attention of researchers. A previous study showed that CDKL5 deficiency interferes with the IQGAP1/cytoplasmic linker protein of 170 kDa (CLIP170)/Rac1 ternary complex formation and negatively influences the microtubule binding of CLIP170 [63]. Furthermore, knockdown of CDKL5 halted cone growth, suppressed axon elongation, and decreased polarised neurons [63, 119]. The neuroactive steroid pregnenolone synthetic derivative, pregnenolone-methyl-ether (also known as 3*β*-methoxy-pregnenolone), has been found to rescue morphological defects in CDKL5-deficient neurons by restoring the microtubule association of CLIP170 [63, 119]. Therefore, targeting the association between CDKL5 and microtubules may be an effective therapeutic strategy.

In conclusion, CDKL5 is strongly related to the pathology of neurodevelopmental disorders. Detailed knowledge of its activity will promote the development of new therapeutic approaches to CDD and further elucidate the mechanisms underlying neuronal development and function. Therefore, an in-depth investigation into CDKL5 is warranted to establish an effective treatment strategy for CDD.

## Figures and Tables

**Table 1:** Substrates of CDKL5.

Substrate	Localisation	Phosphorylation site(s)	Detection of phosphorylated protein	CDKL5 form	References
MeCP2	Nucleus	Not determined	*In vitro*	Full length and catalytic	[40, 42]
Dnmt1	Nucleus	Not determined	*In vitro*	Catalytic	[49]
NGL-1	Plasma membrane	RMNS^631^KDN	*In vitro*	Full length	[38]
Amph1	Cytoplasm	*Mus musculus*: RPRS^293^PSQ *Danio rerio*: RPTS^285^PGP and RPKS^293^PSQ	*In vitro*	Full length and catalytic	[32, 44, 78]
HDAC4	Cytoplasm and nucleus	RAQS^632^SPA	*In vitro*	Full length	[39]
MAP1S	Cytoplasm	*Mus musculus*: RPSS^786^ASA and RPLS^812^ARS *Homo sapiens*: RPLS^900^ARS	*In vitro* and *in vivo*	Full length	[70, 71]
CEP131	Cytoplasm (centrosome)	RPGS^35^AAT	*In vitro*	Full length	[70]
DLG5	Cytoplasm	RPKS^1115^APS	*In vitro*	Full length	[70]
ARHGEF2	Cytoplasm	RPTS^122^AIY	*In vitro*	Full length	[71]
EB2	Cytoplasm	RPSS^222^AKR	*In vitro* and *in vivo*	Full length	[71]
SMAD3	Cytoplasm and nucleus	Not determined	*In vitro*	Full length	[77]

## Data Availability

The following amino acid sequences of the catalytic domain of human protein kinases were used for the phylogenetic tree construction: AMPK*α*1 (accession no. XP_016865113.1), B-Raf (accession no. NP_004324.2), CaMKII*α* (accession no. NP_001350918.1), CDK5 (accession no. CAG33322.1), CDKL1 (accession no. NP_004187.2), CDKL2 (accession no. NP_003939.1), CDKL3 (accession no. NP_001107047.1), CDKL4 (accession no. NP_001333840.1), CDKL5 (accession no. NP_001032420.1), CK1*α* (accession no. NP_001883.4), ERK2 (accession no. NP_002736.3), GSK-3*β* (accession no. NP_002084.2), LRRK2 (accession no. NP_940980.4), MEK1 (accession no. NP_002746.1), MST1 (accession no. AAA83254.1), PKA*α* (accession no. NP_002721.1), p70S6K (accession no. NP_003152.1), and VRK2 (accession no. AAO73049.1). The following sequences were used for the schematic illustration of the primary structure: human CDKL5 (accession no. NP_001310218.1), mouse CDKL5 (mCDKL5_1 in Hector et al. 2016 [29]), zebrafish CDKL5 (accession no. NP_001124243.1), and human ERK2 (accession no. NP_002736.3).

## References

[1] Zhou W., Ercan D., Chen L. (2009). Novel mutant-selective EGFR kinase inhibitors against EGFR T790M. *Nature*.

[2] Moutinho C., Mateus A. R., Milanezi F., Carneiro F., Seruca R., Suriano G. (2008). Epidermal growth factor receptor structural alterations in gastric cancer. *BMC Cancer*.

[3] Song W. J., Sternberg L. R., Kasten-Sportès C. (1996). Isolation of human and murine homologues of the Drosophila minibrain gene: human homologue maps to 21q22.2 in the Down syndrome "critical region". *Genomics*.

[4] Nakano-Kobayashi A., Awaya T., Kii I. (2017). Prenatal neurogenesis induction therapy normalizes brain structure and function in Down syndrome mice. *Proceedings of the National Academy of Sciences of the United States of America*.

[5] Rett A. (1966). On an unusual brain atrophy syndrome in hyperammonemia in childhood. *Wiener Medizinische Wochenschrift (1946)*.

[6] Amir R. E., Van den Veyver I. B., Wan M., Tran C. Q., Francke U., Zoghbi H. Y. (1999). Rett syndrome is caused by mutations in X-linked MECP2, encoding methyl-CpG-binding protein 2. *Nature Genetics*.

[7] Weaving L. S., Christodoulou J., Williamson S. L. (2004). Mutations of CDKL5 cause a severe neurodevelopmental disorder with infantile spasms and mental retardation. *American Journal of Human Genetics*.

[8] Ariani F., Hayek G., Rondinella D. (2008). FOXG1 is responsible for the congenital variant of Rett syndrome. *American Journal of Human Genetics*.

[9] Neul J. L., Kaufmann W. E., Glaze D. G. (2010). Rett syndrome: revised diagnostic criteria and nomenclature. *Annals of Neurology*.

[10] Hadzsiev K., Polgar N., Bene J. (2011). Analysis of Hungarian patients with Rett syndrome phenotype for MECP2, CDKL5 and FOXG1 gene mutations. *Journal of Human Genetics*.

[11] Vidal S., Brandi N., Pacheco P. (2019). The most recurrent monogenic disorders that overlap with the phenotype of Rett syndrome. *European Journal of Paediatric Neurology*.

[12] Mangatt M., Wong K., Anderson B. (2016). Prevalence and onset of comorbidities in the CDKL5 disorder differ from Rett syndrome. *Orphanet Journal of Rare Diseases*.

[13] Demarest S. T., Olson H. E., Moss A. (2019). CDKL5 deficiency disorder: relationship between genotype, epilepsy, cortical visual impairment, and development. *Epilepsia*.

[14] Pringsheim M., Mitter D., Schröder S. (2019). Structural brain anomalies in patients with FOXG1 syndrome and in Foxg1+/- mice. *Annals of Clinical Translational Neurology*.

[15] Fehr S., Wilson M., Downs J. (2013). The CDKL5 disorder is an independent clinical entity associated with early- onset encephalopathy. *European Journal of Human Genetics*.

[16] Tarquinio D. C., Hou W., Berg A. (2017). Longitudinal course of epilepsy in Rett syndrome and related disorders. *Brain*.

[17] Florian C., Bahi-Buisson N., Bienvenu T. (2012). FOXG1-related disorders: from clinical description to molecular genetics. *Molecular Syndromology*.

[18] Vegas N., Cavallin M., Maillard C. (2018). Delineating FOXG1 syndrome: From congenital microcephaly to hyperkinetic encephalopathy. *Neurology Genetics*.

[19] Montini E., Andolfi G., Caruso A. (1998). Identification and characterization of a novel serine-threonine kinase gene from the Xp22 region. *Genomics*.

[20] Kalscheuer V. M., Tao J., Donnelly A. (2003). Disruption of the serine/threonine kinase 9 gene causes severe X-linked infantile spasms and mental retardation. *American Journal of Human Genetics*.

[21] Tao J., Van Esch H., Hagedorn-Greiwe M. (2004). Mutations in the X-linked cyclin-dependent kinase-like 5 (CDKL5/STK9) gene are associated with severe neurodevelopmental retardation. *American Journal of Human Genetics*.

[22] Nectoux J., Heron D., Tallot M., Chelly J., Bienvenu T. (2006). Maternal origin of a novel C-terminal truncation mutation in CDKL5 causing a severe atypical form of Rett syndrome. *Clinical Genetics*.

[23] Sprovieri T., Conforti F. L., Fiumara A. (2009). A novel mutation in the X-linked cyclin-dependent kinase-like 5 (CDKL5) gene associated with a severe Rett phenotype. *American Journal of Medical Genetics Part A*.

[24] Russo S., Marchi M., Cogliati F. (2009). Novel mutations in the CDKL5 gene, predicted effects and associated phenotypes. *Neurogenetics*.

[25] Christianto A., Katayama S., Kameshita I., Inazu T. (2016). A novel CDKL5 mutation in a Japanese patient with atypical Rett syndrome. *Clinica Chimica Acta*.

[26] Fazzari M., Frasca A., Bifari F., Landsberger N. (2019). Aminoglycoside drugs induce efficient read-through of CDKL5 nonsense mutations, slightly restoring its kinase activity. *RNA Biology*.

[27] Yennawar M., White R. S., Jensen F. E. (2019). AMPA receptor dysregulation and therapeutic interventions in a mouse model of CDKL5 deficiency disorder. *The Journal of Neuroscience*.

[28] Manning G., Whyte D. B., Martinez R., Hunter T., Sudarsanam S. (2002). The protein kinase complement of the human genome. *Science*.

[29] Hector R. D., Dando O., Landsberger N. (2016). Characterisation of CDKL5 transcript isoforms in human and mouse. *PLoS One*.

[30] Williamson S. L., Giudici L., Kilstrup-Nielsen C. (2012). A novel transcript of cyclin-dependent kinase-like 5 (CDKL5) has an alternative C-terminus and is the predominant transcript in brain. *Human Genetics*.

[31] Fichou Y., Nectoux J., Bahi-Buisson N., Chelly J., Bienvenu T. (2011). An isoform of the severe encephalopathy-related CDKL5 gene, including a novel exon with extremely high sequence conservation, is specifically expressed in brain. *Journal of Human Genetics*.

[32] Katayama S., Senga Y., Oi A. (2016). Expression analyses of splice variants of zebrafish cyclin-dependent kinase- like 5 and its substrate, amphiphysin 1. *Gene*.

[33] Vitorino M., Cunha N., Conceição N., Cancela M. L. (2018). Expression pattern of cdkl5 during zebrafish early development: implications for use as model for atypical Rett syndrome. *Molecular Biology Reports*.

[34] Fahmi M., Yasui G., Seki K. (2019). In silico study of Rett syndrome treatment-related genes, MECP2, CDKL5, and FOXG1, by evolutionary classification and disordered region assessment. *International Journal of Molecular Sciences*.

[35] Rusconi L., Salvatoni L., Giudici L. (2008). CDKL5 expression is modulated during neuronal development and its subcellular distribution is tightly regulated by the C-terminal tail. *The Journal of Biological Chemistry*.

[36] Oi A., Katayama S., Hatano N., Sugiyama Y., Kameshita I., Sueyoshi N. (2017). Subcellular distribution of cyclin-dependent kinase-like 5 (CDKL5) is regulated through phosphorylation by dual specificity tyrosine-phosphorylation-regulated kinase 1A (DYRK1A). *Biochemical and Biophysical Research Communications*.

[37] Fuchs C., Trazzi S., Torricella R. (2014). Loss of CDKL5 impairs survival and dendritic growth of newborn neurons by altering AKT/GSK-3*β* signaling. *Neurobiology of Disease*.

[38] Ricciardi S., Ungaro F., Hambrock M. (2012). CDKL5 ensures excitatory synapse stability by reinforcing NGL-1-PSD95 interaction in the postsynaptic compartment and is impaired in patient iPSC-derived neurons. *Nature Cell Biology*.

[39] Trazzi S., Fuchs C., Viggiano R. (2016). HDAC4: a key factor underlying brain developmental alterations in CDKL5 disorder. *Human Molecular Genetics*.

[40] Mari F., Azimonti S., Bertani I. (2005). CDKL5 belongs to the same molecular pathway of MeCP2 and it is responsible for the early-onset seizure variant of Rett syndrome. *Human Molecular Genetics*.

[41] Lin C., Franco B., Rosner M. R. (2005). CDKL5/Stk9 kinase inactivation is associated with neuronal developmental disorders. *Human Molecular Genetics*.

[42] Bertani I., Rusconi L., Bolognese F. (2006). Functional consequences of mutations in CDKL5, an X-linked gene involved in infantile spasms and mental retardation. *The Journal of Biological Chemistry*.

[43] Lewis J. D., Meehan R. R., Henzel W. J. (1992). Purification, sequence, and cellular localization of a novel chromosomal protein that binds to methylated DNA. *Cell*.

[44] Sekiguchi M., Katayama S., Hatano N., Shigeri Y., Sueyoshi N., Kameshita I. (2013). Identification of amphiphysin 1 as an endogenous substrate for CDKL5, a protein kinase associated with X-linked neurodevelopmental disorder. *Archives of Biochemistry and Biophysics*.

[45] Carouge D., Host L., Aunis D., Zwiller J., Anglard P. (2010). CDKL5 is a brain MeCP2 target gene regulated by DNA methylation. *Neurobiology of Disease*.

[46] Livide G., Patriarchi T., Amenduni M. (2015). GluD1 is a common altered player in neuronal differentiation from both MECP2-mutated and CDKL5-mutated iPS cells. *European Journal of Human Genetics*.

[47] Kameshita I., Tsuge T., Kinashi T. (2003). A new approach for the detection of multiple protein kinases using monoclonal antibodies directed to the highly conserved region of protein kinases. *Analytical Biochemistry*.

[48] Sugiyama Y., Sueyoshi N., Shigeri Y. (2005). Generation and application of a monoclonal antibody that detects a wide variety of protein tyrosine kinases. *Analytical Biochemistry*.

[49] Kameshita I., Sekiguchi M., Hamasaki D. (2008). Cyclin-dependent kinase-like 5 binds and phosphorylates DNA methyltransferase 1. *Biochemical and Biophysical Research Communications*.

[50] Yoder J. A., Soman N. S., Verdine G. L., Bestor T. H. (1997). DNA (cytosine-5)-methyltransferases in mouse cells and tissues. studies with a mechanism-based probe. *Journal of Molecular Biology*.

[51] Sugiyama Y., Hatano N., Sueyoshi N. (2010). The DNA-binding activity of mouse DNA methyltransferase 1 is regulated by phosphorylation with casein kinase 1delta/epsilon. *The Biochemical Journal*.

[52] Lavoie G., Estève P. O., Laulan N. B., Pradhan S., St-Pierre Y. (2011). PKC isoforms interact with and phosphorylate DNMT1. *BMC Biology*.

[53] Nishimura-Akiyoshi S., Niimi K., Nakashiba T., Itohara S. (2007). Axonal netrin-Gs transneuronally determine lamina-specific subdendritic segments. *Proceedings of the National Academy of Sciences of the United States of America*.

[54] Borg I., Freude K., Kübart S. (2005). Disruption of Netrin G1 by a balanced chromosome translocation in a girl with Rett syndrome. *European Journal of Human Genetics*.

[55] Archer H. L., Evans J. C., Millar D. S. (2006). NTNG1 mutations are a rare cause of Rett syndrome. *American Journal of Medical Genetics Part A*.

[56] Nectoux J., Girard B., Bahi-Buisson N. (2007). Netrin G1 mutations are an uncommon cause of atypical Rett syndrome with or without epilepsy. *Pediatric Neurology*.

[57] Senga Y., Nagamine T., Sekiguchi M., Kaneko K., Sueyoshi N., Kameshita I. (2011). Detection of protein kinase substrates in tissue extracts after separation by isoelectric focusing. *Analytical Biochemistry*.

[58] Heuser J. E., Reese T. S. (1973). Evidence for recycling of synaptic vesicle membrane during transmitter release at the frog neuromuscular junction. *The Journal of Cell Biology*.

[59] Takano T., Tomomura M., Yoshioka N. (2012). LMTK1/AATYK1 is a novel regulator of axonal outgrowth that acts via Rab11 in a Cdk5-dependent manner. *The Journal of Neuroscience*.

[60] Wakita Y., Kakimoto T., Katoh H., Negishi M. (2011). The F-BAR protein Rapostlin regulates dendritic spine formation in hippocampal neurons. *The Journal of Biological Chemistry*.

[61] Della Sala G., Putignano E., Chelini G. (2016). Dendritic spine instability in a mouse model of CDKL5 disorder is rescued by insulin-like growth factor 1. *Biological Psychiatry*.

[62] Chen Q., Zhu Y. C., Yu J. (2010). CDKL5, a protein associated with rett syndrome, regulates neuronal morphogenesis via Rac1 signaling. *The Journal of Neuroscience*.

[63] Barbiero I., Peroni D., Tramarin M. (2017). The neurosteroid pregnenolone reverts microtubule derangement induced by the loss of a functional CDKL5-IQGAP1 complex. *Human Molecular Genetics*.

[64] Chen Y., Wang Y., Modrusan Z., Sheng M., Kaminker J. S. (2014). Regulation of neuronal gene expression and survival by basal NMDA receptor activity: a role for histone deacetylase 4. *The Journal of Neuroscience*.

[65] Li J., Chen J., Ricupero C. L. (2012). Nuclear accumulation of HDAC4 in ATM deficiency promotes neurodegeneration in ataxia telangiectasia. *Nature Medicine*.

[66] Bolger T. A., Yao T. P. (2005). Intracellular trafficking of histone deacetylase 4 regulates neuronal cell death. *The Journal of Neuroscience*.

[67] D'Mello S. R. (2009). Histone deacetylases as targets for the treatment of human neurodegenerative diseases. *Drug News & Perspectives*.

[68] Katayama S., Morii A., Makanga J. O., Suzuki T., Miyata N., Inazu T. (2018). HDAC8 regulates neural differentiation through embryoid body formation in P19 cells. *Biochemical and Biophysical Research Communications*.

[69] Saikusa T., Hara M., Iwama K. (2018). De novo HDAC8 mutation causes Rett-related disorder with distinctive facial features and multiple congenital anomalies. *Brain & Development*.

[70] Muñoz I. M., Morgan M. E., Peltier J. (2018). Phosphoproteomic screening identifies physiological substrates of the CDKL5 kinase. *The EMBO Journal*.

[71] Baltussen L. L., Negraes P. D., Silvestre M. (2018). Chemical genetic identification of CDKL5 substrates reveals its role in neuronal microtubule dynamics. *The EMBO Journal*.

[72] Tegha-Dunghu J., Bausch E., Neumann B. (2014). MAP1S controls microtubule stability throughout the cell cycle in human cells. *Journal of Cell Science*.

[73] Xie R., Nguyen S., McKeehan K., Wang F., McKeehan W. L., Liu L. (2011). Microtubule-associated protein 1S (MAP1S) bridges autophagic components with microtubules and mitochondria to affect autophagosomal biogenesis and degradation. *The Journal of Biological Chemistry*.

[74] Xie R., Wang F., McKeehan W. L., Liu L. (2011). Autophagy enhanced by microtubule- and mitochondrion-associated MAP1S suppresses genome instability and hepatocarcinogenesis. *Cancer Research*.

[75] Canning P., Park K., Gonçalves J. (2018). CDKL family kinases have evolved distinct structural features and ciliary function. *Cell Reports*.

[76] Goldspink D. A., Gadsby J. R., Bellett G. (2013). The microtubule end-binding protein EB2 is a central regulator of microtubule reorganisation in apico-basal epithelial differentiation. *Journal of Cell Science*.

[77] Fuchs C., Medici G., Trazzi S. (2019). CDKL5 deficiency predisposes neurons to cell death through the deregulation of SMAD3 signaling. *Brain Pathology*.

[78] Katayama S., Sueyoshi N., Kameshita I. (2015). Critical determinants of substrate recognition by cyclin-dependent kinase-like 5 (CDKL5). *Biochemistry*.

[79] Songyang Z., Lu K. P., Kwon Y. T. (1996). A structural basis for substrate specificities of protein Ser/Thr kinases: primary sequence preference of casein kinases I and II, NIMA, phosphorylase kinase, calmodulin-dependent kinase II, CDK5, and Erk1. *Molecular and Cellular Biology*.

[80] Kameshita I., Taketani S., Ishida A., Fujisawa H. (1999). Detection of a variety of Ser/Thr protein kinases using a synthetic peptide with multiple phosphorylation sites. *Journal of Biochemistry*.

[81] Clark-Lewis I., Sanghera J. S., Pelech S. L. (1991). Definition of a consensus sequence for peptide substrate recognition by p44mpk, the meiosis-activated myelin basic protein kinase. *The Journal of Biological Chemistry*.

[82] Himpel S., Tegge W., Frank R., Leder S., Joost H. G., Becker W. (2000). Specificity determinants of substrate recognition by the protein kinase Dyrk1a. *The Journal of Biological Chemistry*.

[83] Haribabu B., Hook S. S., Selbert M. A. (1995). Human calcium-calmodulin dependent protein kinase I: cDNA cloning, domain structure and activation by phosphorylation at threonine-177 by calcium-calmodulin dependent protein kinase I kinase. *The EMBO Journal*.

[84] Selbert M. A., Anderson K. A., Huang Q. H., Goldstein E. G., Means A. R., Edelman A. M. (1995). Phosphorylation and activation of Ca^2+^ -calmodulin-dependent protein kinase IV by Ca^2+^ -calmodulin-dependent protein kinase Ia kinase. Phosphorylation of threonine 196 is essential for activation. *The Journal of Biological Chemistry*.

[85] Zheng C. F., Guan K. L. (1993). Cloning and characterization of two distinct human extracellular signal-regulated kinase activator kinases, MEK1 and MEK2. *The Journal of Biological Chemistry*.

[86] Ikeda A., Okuno S., Fujisawa H. (1991). Studies on the generation of Ca2+/calmodulin-independent activity of calmodulin-dependent protein kinase II by autophosphorylation. Autothiophosphorylation of the enzyme. *Journal of Biological Chemistry*.

[87] Graves P. R., Roach P. J. (1995). Role of COOH-terminal phosphorylation in the regulation of casein kinase I delta. *The Journal of Biological Chemistry*.

[88] Heist E. K., Srinivasan M., Schulman H. (1998). Phosphorylation at the nuclear localization signal of Ca2+/calmodulin-dependent protein kinase II blocks its nuclear targeting. *The Journal of Biological Chemistry*.

[89] Xiang X., Zang M., Waelde C. A., Wen R., Luo Z. (2002). Phosphorylation of 338SSYY341 regulates specific interaction between Raf-1 and MEK1. *The Journal of Biological Chemistry*.

[90] Katayama S., Sugiyama Y., Hatano N., Terachi T., Sueyoshi N., Kameshita I. (2012). PKL01, an Ndr kinase homologue in plant, shows tyrosine kinase activity. *Journal of Biochemistry*.

[91] Rusconi L., Kilstrup-Nielsen C., Landsberger N. (2011). Extrasynaptic N-methyl-D-aspartate (NMDA) receptor stimulation induces cytoplasmic translocation of the CDKL5 kinase and its proteasomal degradation. *The Journal of Biological Chemistry*.

[92] La Montanara P., Rusconi L., Locarno A. (2015). Synaptic synthesis, dephosphorylation, and degradation: a novel paradigm for an activity-dependent neuronal control of CDKL5. *The Journal of Biological Chemistry*.

[93] Lek M., Karczewski K. J., Minikel E. V. (2016). Analysis of protein-coding genetic variation in 60,706 humans. *Nature*.

[94] Landrum M. J., Lee J. M., Benson M. (2018). ClinVar: improving access to variant interpretations and supporting evidence. *Nucleic Acids Research*.

[95] Katayama S., Inazu T. (2019). Straightforward and rapid method for detection of cyclin-dependent kinase-like 5 activity. *Analytical Biochemistry*.

[96] Krishnaraj R., Ho G., Christodoulou J. (2017). RettBASE: Rett syndrome database update. *Human Mutation*.

[97] Wang I. T., Allen M., Goffin D. (2012). Loss of CDKL5 disrupts kinome profile and event-related potentials leading to autistic-like phenotypes in mice. *Proceedings of the National Academy of Sciences of the United States of America*.

[98] Amendola E., Zhan Y., Mattucci C. (2014). Mapping pathological phenotypes in a mouse model of CDKL5 disorder. *PLoS One*.

[99] Sivilia S., Mangano C., Beggiato S. (2016). CDKL5 knockout leads to altered inhibitory transmission in the cerebellum of adult mice. *Genes, Brain, and Behavior*.

[100] Lo Martire V., Alvente S., Bastianini S. (2017). CDKL5 deficiency entails sleep apneas in mice. *Journal of Sleep Research*.

[101] Mazziotti R., Lupori L., Sagona G. (2017). Searching for biomarkers of CDKL5 disorder: early-onset visual impairment in CDKL5 mutant mice. *Human Molecular Genetics*.

[102] Tang S., Wang I. J., Yue C. (2017). Loss of CDKL5 in glutamatergic neurons disrupts hippocampal microcircuitry and leads to memory impairment in mice. *The Journal of Neuroscience*.

[103] Jhang C. L., Huang T. N., Hsueh Y. P., Liao W. (2017). Mice lacking cyclin-dependent kinase-like 5 manifest autistic and ADHD-like behaviors. *Human Molecular Genetics*.

[104] Okuda K., Takao K., Watanabe A., Miyakawa T., Mizuguchi M., Tanaka T. (2018). Comprehensive behavioral analysis of the Cdkl5 knockout mice revealed significant enhancement in anxiety- and fear-related behaviors and impairment in both acquisition and long-term retention of spatial reference memory. *PLoS One*.

[105] Fuchs C., Gennaccaro L., Trazzi S. (2018). Heterozygous CDKL5 knockout female mice are a valuable animal model for CDKL5 disorder. *Neural Plasticity*.

[106] Okuda K., Kobayashi S., Fukaya M. (2017). CDKL5 controls postsynaptic localization of GluN2B-containing NMDA receptors in the hippocampus and regulates seizure susceptibility. *Neurobiology of Disease*.

[107] Fuchs C., Rimondini R., Viggiano R. (2015). Inhibition of GSK3*β* rescues hippocampal development and learning in a mouse model of CDKL5 disorder. *Neurobiology of Disease*.

[108] Fuchs C., Fustini N., Trazzi S., Gennaccaro L., Rimondini R., Ciani E. (2018). Treatment with the GSK3-beta inhibitor Tideglusib improves hippocampal development and memory performance in juvenile, but not adult, Cdkl5 knockout mice. *The European Journal of Neuroscience*.

[109] Jorge-Torres O. C., Szczesna K., Roa L. (2018). Inhibition of Gsk3b reduces Nfkb1 signaling and rescues synaptic activity to improve the Rett syndrome phenotype in Mecp2-knockout mice. *Cell Reports*.

[110] Castro J., Garcia R. I., Kwok S. (2014). Functional recovery with recombinant human IGF1 treatment in a mouse model of Rett syndrome. *Proceedings of the National Academy of Sciences of the United States of America*.

[111] Vestling M., Wiehager B., Tanii H., Cowburn R. F. (2001). Akt activity in presenilin 1 wild-type and mutation transfected human SH-SY5Y neuroblastoma cells after serum deprivation and high glucose stress. *Journal of Neuroscience Research*.

[112] Jones D. M., Tucker B. A., Rahimtula M., Mearow K. M. (2003). The synergistic effects of NGF and IGF-1 on neurite growth in adult sensory neurons: convergence on the PI 3-kinase signaling pathway. *Journal of Neurochemistry*.

[113] Zhu Y. C., Li D., Wang L. (2013). Palmitoylation-dependent CDKL5-PSD-95 interaction regulates synaptic targeting of CDKL5 and dendritic spine development. *Proceedings of the National Academy of Sciences of the United States of America*.

[114] Zhang Y., Matt L., Patriarchi T. (2014). Capping of the N-terminus of PSD-95 by calmodulin triggers its postsynaptic release. *The EMBO Journal*.

[115] Fuchs C., Gennaccaro L., Ren E. (2020). Pharmacotherapy with sertraline rescues brain development and behavior in a mouse model of CDKL5 deficiency disorder. *Neuropharmacology*.

[116] Barbiero I., Valente D., Chandola C. (2017). CDKL5 localizes at the centrosome and midbody and is required for faithful cell division. *Scientific Reports*.

[117] Fehr S., Downs J., Ho G. (2016). Functional abilities in children and adults with the CDKL5 disorder. *American Journal of Medical Genetics Part A*.

[118] Fehr S., Wong K., Chin R. (2016). Seizure variables and their relationship to genotype and functional abilities in the CDKL5 disorder. *Neurology*.

[119] Barbiero I., Peroni D., Siniscalchi P. (2020). Pregnenolone and pregnenolone-methyl-ether rescue neuronal defects caused by dysfunctional CLIP170 in a neuronal model of CDKL5 deficiency disorder. *Neuropharmacology*.

